# Worldwide literature on epidemiology of human alveolar echinococcosis: a systematic review of research published in the twenty-first century

**DOI:** 10.1007/s15010-019-01325-2

**Published:** 2019-05-30

**Authors:** Sven Baumann, Rong Shi, Wenya Liu, Haihua Bao, Julian Schmidberger, Wolfgang Kratzer, Weixia Li, the interdisciplinary Echinococcosis Working Group Ulm, Thomas F. E. Barth, Sven Baumann, Johannes Bloehdorn, Iris Fischer, Tilmann Graeter, Natalja Graf, Beate Gruener, Doris Henne-Bruns, Andreas Hillenbrand, Tanja Kaltenbach, Peter Kern, Petra Kern, Katharina Klein, Wolfgang Kratzer, Niloofar Ehteshami, Patrycja Schlingeloff, Julian Schmidberger, Rong Shi, Yael Staehelin, Frauke Theis, Daniil Verbitskiy, Ghaith Zarour

**Affiliations:** 1 Department of Internal Medicine I, Ulm University Hospital, Albert-Einstein-Allee 23, 89081 Ulm, Germany; 2 Department of Diagnostic and Interventional Radiology, Ulm University Hospital, Albert-Einstein-Allee 23, 89081 Ulm, Germany; 3 Xinjiang Medical University, First Affiliated Hospital, WHO Collaborating Centre on Prevention and Care Management of Echinococcosis, Urumqi, 830000 Xinjiang Uyghur Autonomous Region People’s Republic of China; 4 Qinghai University Affiliated Hospital, Qinghai University, Xining, 810001 Qinghai People’s Republic of China

**Keywords:** Alveolar echinococcosis, *Echinococcus multilocularis*, Worldwide epidemiology, Geographical distribution, Prevalence, Maps

## Abstract

**Purpose:**

Human alveolar echinococcosis (AE) is a potentially lethal zoonosis caused by the cestode *Echinococcus multilocularis*. The aim of this systematic review is to establish a comprehensive global AE literature overview taking into account the epidemiologically relevant AE research of the twenty-first century.

**Methods:**

We systematically searched the global literature published from 2001 through 2018 via MEDLINE, EMBASE, the Russian databases eLIBRARY.RU, CyberLeninka, the Chinese databases CNKI, VIP, Journals.research.ac.ir (Farsi language-based), Jordan E-Library (Arab language-based) and supplementary Google Scholar, in accordance with the PRISMA guidelines. QGIS software was used for the mapping of the affected countries.

**Results:**

We have listed 154 relevant publications in the final literature synopsis in consideration of our quality assessment. Including non-autochthonous cases, human AE was reported in 36 countries within the northern hemisphere from 2001 to 2018. The first publication of AE in Tajikistan, Pakistan, South Korea, Belgium, the Netherlands, Slovakia, Hungary, Lithuania, Latvia, Slovenia and Morocco occurred in this century; further first cases in Taiwan, Thailand, and Denmark were considered to be non-autochthonous by the authors. The highest total case numbers (*n* ≥ 100 in a single article) were reported in France, Germany, Switzerland, Poland, and Lithuania, including China and Kyrgyzstan with by far the highest prevalence figures.

**Conclusions:**

Our paper emphasises the increasing spread of reported cases and the rise in its numbers in the literature of the twenty-first century, especially in western, northern and eastern Europe, as well as in central Asia. Epidemiological studies on human infections are lacking in many parts of the world.

## Introduction

Human alveolar echinococcosis (AE) is a rare, life-threatening zoonosis caused by the larvae of *Echinococcus multilocularis* (*E. multilocularis*), a helminth of the Cestoda class. Transmission is through ingestion of parasite eggs, which are excreted in the faeces of the definitive host. The life cycle of *E. multilocularis* takes place between canids as the definitive hosts and their prey, small mammals such as rodents, which act as intermediate hosts [1]. Besides the original cycles in wild animals [red foxes (*Vulpes vulpes*) and voles being the most important in Europe], cycles also seem to have become established in domestic dogs (*Canis lupus familiaris*) [2]. In the Chinese province of Ningxia, for example, wild canids are virtually non-existent and dogs are the most significant transmitters of AE [2]. Humans are accidental intermediate hosts. In 98% of cases, infection manifests primarily in the liver, showing a tumour-like malignant growth which, left untreated, leads to death in 90% of cases within 10–15 years of diagnosis [3, 4]. Annually there are estimated more than 18,000 new cases worldwide of AE, with 91% of those occurring in China [5].

Corresponding to the hazardous nature of the disease, WHO has designated AE as 1 of the 20 neglected tropical diseases and *E. multilocularis* as the food-borne parasite with the third largest global impact of 24 ranked parasites [6, 7].

*Echinococcus multilocularis* is found throughout the animal world in moderate to cold climate zones in the northern hemisphere. It extends from western, northern and eastern Europe and Russia into Asia, from eastern Turkey across central Asia into western and northern China, and is endemic on the northern Japanese island of Hokkaido. In North America, the helminth is endemic to the northwest coastal areas of Alaska, the western Canadian Arctic, southern Canada, and the neighbouring central northern states of the USA [8]. Cases of human disease do not necessarily occur in all endemic areas. The different rates of parasitic infection observed in the wildlife compared with the spread of human AE rest on various factors, such as host-dependant transmission patterns, landscape characteristics such as grass lands, local socioeconomic conditions including awareness of the disease within the public health system and general population [9–11]. A further deciding factor is thought to be the considerable variation in the intraspecific human pathogenicity of the parasite and the human host susceptibility [2]. Analyses of the genetic diversity of *E. multilocularis* have already demonstrated variants of the so-called Asian, European, North American, and Mongolian strains [12–14].

This review article is intended to provide the basis for a literature synopsis on the prevalence of AE worldwide. This should help to depict the spread of the disease across the globe, demonstrate current trends, and reveal gaps in our epidemiological knowledge. Furthermore, a global map focussing only on human cases should give an overview in which countries AE has been described in the current literature.

## Methods

### Search strategy and selection criteria

We performed a systematic literature search for worldwide relevant publications in the bibliographical databases MEDLINE (via the PubMed metasearch engine), EMBASE (via the OVID metasearch engine), the Chinese databases CNKI, VIP, and the Russian Scientific Electronic Library (via eLIBRARY.RU). These searches were supplemented with the Russian open access repository CyberLeninka, the Farsi language-based database Journals.research.ac.ir, the Arab language-based database of the University of Jordan E-Library and the web search engine Google Scholar.

The countries for our area-specific search strategy were selected after an initial screening of general reviews on *E. multilocularis* distribution, and its neighbouring nations. All the internationally relevant keywords for the disease were linked with the Boolean operator “OR”. The search key was designed to be as narrow as possible to ensure goal-oriented results but at the same time broad enough to capture all the relevant world literature. Search key optimisation was carried out by analysing the search details of each search term and subsequent pilot testing. The width of the search came primarily through automatic term mapping, the automatic generation of a more detailed search string, which also covered Medical Subject Headings (MeSH) terms (e.g. the MeSH term “*Echinococcus multilocularis*”). MeSH is the controlled vocabulary thesaurus generated in MEDLINE. A similar procedure was carried out with EMBASE (Emtree). The Boolean operator “AND” was used to add the country to be screened and the corresponding adjective, as well as any possible ethnonym, superordinate region (e.g. “Slavic” or “Baltic”) or subordinate region (e.g. “Alaska”, “Tibet”) to the end of the general search string. This resulted in PubMed search keys such as the one for France:* Echinococcus multilocularis* OR echinococcus alveolaris OR alveolar hydatid disease OR alveolar hydatid cyst OR alveolar hydatidosis OR alveococcosis AND (France OR French). Truncation, double quotes, and also the search term “alveolar echinococcosis” did not lead to a higher number of search results. Alongside the terms in Latin letters, synonyms in Chinese, Cyrillic and Arabic script were also searched through the suitable data bases.

The search was restricted to articles that were published from 2001 to 2018. There were neither restrictions in terms of language, place of publication, nor the time of the initial AE diagnosis; therefore, it can be dated before 2001. Overall case numbers included non-autochthonous cases, which were given in parenthesis in the final synopsis (e.g. a total of 65 cases, two of which were considered non-autochthonous, was given as “65 (2) cases”). With respect to the assumed infection locality, we used the information given by the authors. The searches were carried out between 01 June 2017 and 15 October 2017, February 2018, and between 14 January and 15 March 2019.

In order to be included in the final literature list, we established a quality assessment following previously defined including and excluding criteria for the collected data.

Inclusion criteria:

1. The article concerned epidemiological data on human AE (case numbers, prevalence, incidence) including transparent units (e.g. the incidence given as the number of cases per 100,000 inhabitants per year).
2. The data arose from clearly documented diagnostic criteria [serology, ultrasound (US), computed tomography (CT), magnetic resonance imaging (MRI), histopathology, nucleic acid-based testing] or from an official registry.

Exclusion criteria:

1. Articles in governmental publications for public health monitoring, or data that came from another study, reports of non-governmental organisations, congress contributions, or opinions of expert committees.
2. Data that were based on serological investigations without additional imaging.
3. No distinction was made between AE and cystic echinococcosis (CE).
4. Data that were interpolated or estimated.
5. Data that did not represent the basic population of AE cases, meaning certain preselected groups with no relation to a larger population size, or that came from a case report (with the exception of articles reporting cases in countries where no studies from 2001 to 2018 could be found with case numbers of *n* ≥ 10).

Qualification for the final literature list was carried out in two steps. First, we inspected all the articles found in the search results and applied the defined criteria. Then, we looked closely at all references that appeared relevant in each article. All the literature then discovered was inspected in the same way. We repeated the procedure until no more relevant information was generated (snowball method). The data obtained were stored in an Excel table (Microsoft Office 2017, version 15.30) and divided into the following categories: country, subordinate region (administrative unit), paper (ID, lead author, year of publication, title, journal, volume, pages), period of data collection, epidemiological data (case numbers, prevalence, incidence), case definition (serology, US, CT, MRI, histopathology, nucleic acid-based testing), and non-autochthonous cases.

A multilinguistic team of researchers screened the articles. Two researchers (SB, physician; JS, epidemiologist) independently inspected the literature according to the inclusion and exclusion criteria. In general, the full article was screened, unless the abstract was not clearly leading to an exclusion (e.g. the article was only about CE cases). All non-English articles were analysed in cooperation with native speakers. Literature in the Chinese language was independently screened by WXL, physician and RS, physician. Any uncertainties about the inclusion of an article were discussed, and if a consensus was not met, WK, physician, and HB, physician, were consulted to obtain it. An attempt was made to contact the corresponding author whenever there were any unresolved questions regarding the period of data collection or the case definition.

We used QGIS software (version 2.18.21) to generate the world map. Each country in which cases of AE had been reported in the literature between 2001 and 2018 was mapped. For the topographical colour shading of a country, the highest total number of cases in one reference within this period was the deciding factor.

This systematic overview follows the Preferred Reporting Items for Systematic Reviews and Meta-Analyses (PRISMA) guidelines to ensure a transparent study [15]. The corresponding checklist is attached as supporting information. The search protocol was entered into the International Prospective Register of Systematic Reviews (PROSPERO) under Registration number CRD42017079097.

## Results

**Fig. 1:**
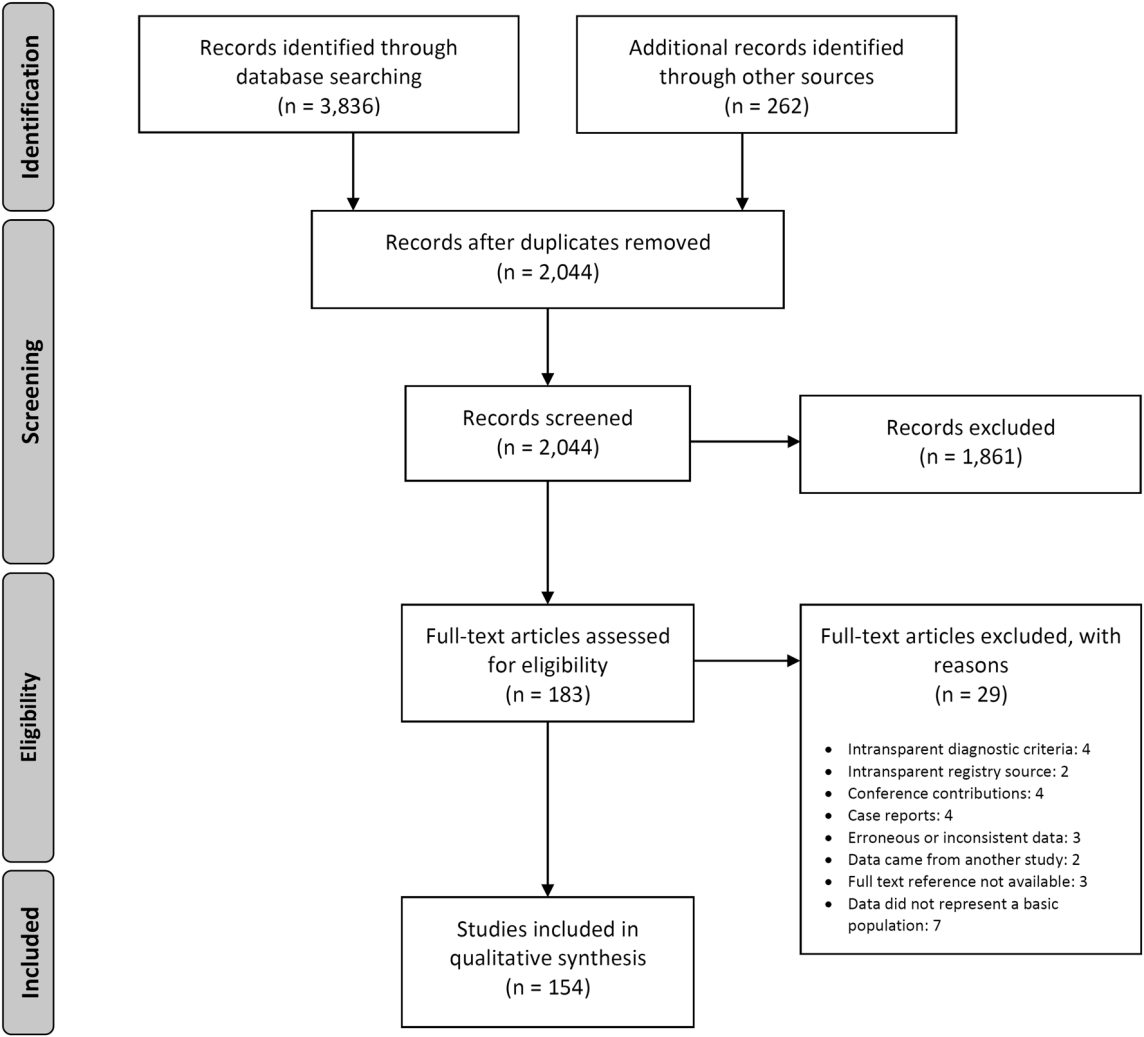
Following the PRISMA guidelines, the flowchart represents the algorithm for article selection

**Fig. 2:**
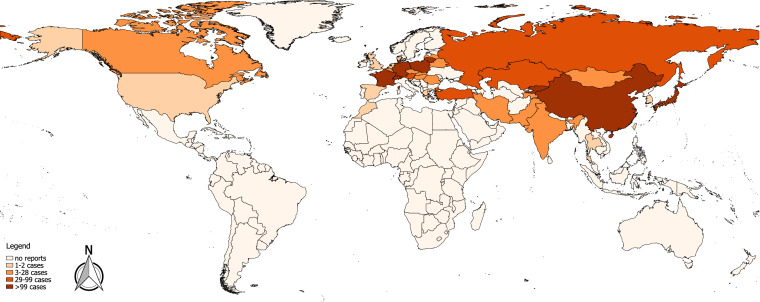
Worldwide distribution of alveolar echinococcosis in humans according to the published literature 2001–2018. Each country in which cases of AE had been reported in the literature between 2001 and 2018 was mapped. For the topographical colour shading of a country, the highest total number of cases in one reference within this period was the deciding factor

**Fig. 3:**
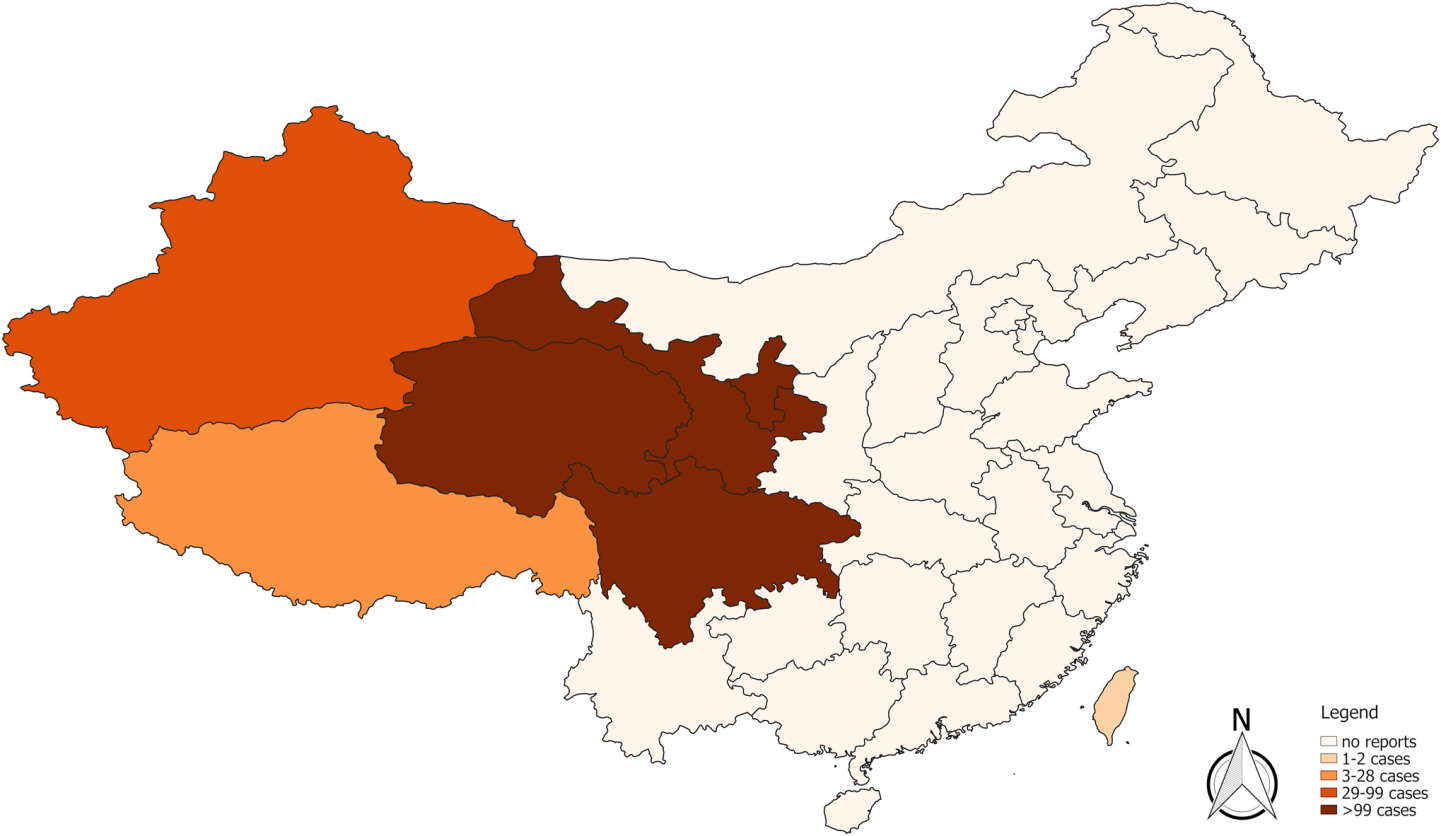
Current distribution of alveolar echinococcosis in humans according to the published literature 2001–2018 in China. Each province in which cases of AE had been reported in the literature between 2001 and 2018 was mapped. For the topographical colour shading of a province, the highest total number of cases in one reference within this period was the deciding factor

The numerical results of our search for the worldwide literature on AE through MEDLINE, EMBASE, the Chinese databases CNKI, VIP, Russian Scientific Electronic Library, CyberLeninka, Journals.research.ac.ir, Jordan E-Library and Google Scholar are presented as a flowchart in Fig. 1. Overall, we screened 99 countries or national territories independently, for potentially relevant publications. Relevant sources were found in 75 countries (*n* = 3836). A further 262 articles were detected in the course of our research, as relevant citations in the literature originally inspected (snowball method), giving a total of 4098 identified articles. We eliminated any duplicates in the various databases found with the country-specific key. Of the 2044 publications now under consideration, 1861 were excluded on inspection, as they obviously did not meet our requirements. The 183 remaining articles were then examined in detail with respect to the inclusion and exclusion criteria, where 29 further publications were excluded. Therefore, the final number of references was 154 (Tables 1, 2, 3). Figure 2 portrays the world map of all the affected countries and Fig. 3 depicts all the involved Chinese provinces.

**Table 1:** Asia

Country	Region	Total case number (*n*)	Prevalence (*n*/10^5^)	Incidence (*n*/10^5^/year)	Period covered by data	Particulars of the population	S	I	US	CT	MRI	HP	DNA	References
China	Gansu (Zhang and Min Counties)	84	3400		1994–1997	Han Chinese population	(+)	+	+	n/a	n/a	n/a	n/a	Bartholomot [126], Shi [127]
	Gansu	119^b^	3390		1996–1997, 2003		(+)	(+)	(+)	n/a	n/a	n/a	n/a	Shi [36]
	Gansu (Gannan Tibetan Autonomous Prefecture)	3	1		2007–2013		(+)	+	+	n/a	n/a	n/a	n/a	Ma [128], Wang [129]
	Gansu (Minle County)	9^b^			n/a	Han Chinese population	(+)	(+)	(+)	(+)	n/a	(+)	(+)	Han [130]
	Gansu	1	450		Sep 2011–Jun 2012, 2017	Tibetan rural population	+	+	+	n/a	n/a	n/a	n/a	Wang [131]
	Ningxia	263			1985–2001		(+)	+	+	(+)	n/a	(+)	n/a	Li [132]
	Ningxia	11	1730		2002		+	+	+	n/a	n/a	n/a	n/a	Li [133]
	Ningxia (Xiji, Guayan, Haiyuan Counties)	82	3700		1985–2001		n/a	(+)	(+)	(+)	n/a	(+)	n/a	Yang [35]
	Ningxia (Xiji County)	9 ^g^			2001–2002	Hui rural population (113 inhabitants of Nanwan village)^g^	(+)	+	+	n/a	n/a	n/a	n/a	Yang [134]
	Ningxia	96	2000		2002–2003		(+)	+	+	n/a	n/a	n/a	n/a	Yang [135]
	Ningxia	79	2200		2002	Schoolchildren, 7–18 years	(+)	+	+	n/a	n/a	n/a	n/a	Yang [87], Yang [135]
	Ningxia	96	3000		2002–2003	Non-student subset of data of [135]	(+)	+	+	n/a	n/a	n/a	n/a	Pleydell [136], Yang [135]
	Ningxia (Xiji County)	15			2006–2007	Children/Adolescents, 6–20 years	(+)	+	+	n/a	n/a	n/a	n/a	Fang [137]
	Qinghai (Xinghai County)	1	160		Jun–Jul 1999		+	+	+	n/a	n/a	n/a	n/a	Wu [138]
	Qinghai and Sichuan	108	1400		Jun 1997–Aug 1998		+	+	+	n/a	n/a	n/a	n/a	Qiu [139]
	Qinghai (Chindu, Zeko, Gade Counties)	31	800		1997–1998		+	+	+	n/a	n/a	n/a	n/a	Schantz [140]
	Qinghai (Yushu County)	4	500		2001		+	+	+	n/a	n/a	n/a	n/a	He [141]
	Qinghai	125	1910		1995–2005		+	+	+	n/a	n/a	n/a	n/a	Wang [142]
	Qinghai (Jiuzhi County)	39	2250		Sep–Oct 2005		+	+	+	n/a	n/a	n/a	n/a	Wu [143]
	Qinghai (Zhiduo County)		200		2006		(+)	+	+	n/a	n/a	n/a	n/a	Wu [144]
	Qinghai (Jiuzhi County)	39	2500		2005	Tibetan population	(+)	+	+	n/a	n/a	n/a	n/a	Yu [145]
	Qinghai	141	8200		Aug–Sep 2007		+	+	+	n/a	n/a	n/a	n/a	Han [146]
	Qinghai	114	1000		1990–2010	Children, 6–15 years/Tibetan rural population	+	+	+	n/a	n/a	n/a	n/a	Cai [147]
	Qinghai	114	600		2000–2010	Children, 6–15 years	(+)	+	+	n/a	n/a	n/a	n/a	Cai [148]
	Qinghai and Sichuan	577	3700		2002–2008		(+)	+	+	n/a	n/a	n/a	n/a	Giraudoux [149]
	Qinghai	17			2006–2014^a^		n/a	n/a	n/a	n/a	n/a	n/a	+	Ma [150]
	Qinghai (Maqên County)	34	2200		n/a		(+)	+	+	n/a	n/a	n/a	n/a	Ma [151]
	Qinghai (Banma County)	170	9430		Jul–Aug 2014		+	+	+	n/a	n/a	n/a	n/a	Ren [17]
	Qinghai (Banma and Dari Counties)	16	1670		2015	R; Children, 3–17 years	+	+	+	n/a	n/a	n/a	n/a	Hou [152]
	Qinghai (Hainan Tibetan Autonomous Prefecture)	1	2780		2016	Tibetan rural population	+	+	+	n/a	n/a	n/a	n/a	Cai [153]
	Quinghai (Maqin, Gander, Dari, Jiuzhi, Banma Counties)	146	1300		2011	Schoolchildren, 6–16 years	+	+	+	n/a	n/a	n/a	n/a	Cai [86]
	Qinghai (Yushu and Guoluo Prefectures)	221	1130		2012–2014	Children, 6–12 years/Tibetan rural population	+	+	+	n/a	n/a	n/a	n/a	Han [154]
	Qinghai (Huangnan Prefecture)	29	1150		2012–2014, 2017	Tibetan rural population	+	+	+	n/a	n/a	n/a	n/a	Niang [155]
	Qinghai	222	1100		2010–2011	Schoolchildren, 6–18 years	(+)	+	+	n/a	n/a	n/a	n/a	Han [156]
	Sichuan (Shiqu County)	60	8500		2001–2002	Village-based study population	n/a	+	+	n/a	n/a	n/a	n/a	Wang [31]
	Sichuan (Shiqu County)	180	5740		2001–2003		n/a	+	+	n/a	n/a	n/a	n/a	Budke [157]
	Sichuan (Shiqu County)	198	6200		2000–2002	Village-based study population	(+)	+	+	n/a	n/a	n/a	n/a	Li [158]
	Sichuan (Baiyü, Seda, Batang, Litang Counties)	37	1230		2002–2003		(+)	+	+	n/a	n/a	n/a	n/a	Yu [159]
	Sichuan	85	2540		2004–2005		n/a	+	+	n/a	n/a	n/a	n/a	Renqingpengcuo [160]
	Sichuan (Ganzi and Shiqu Counties)	223	3100		1997, 2001, 2002, 2003		+	+	+	n/a	n/a	n/a	n/a	Wang [161]
	Sichuan	311	3050		2001–2008		(+)	+	+	n/a	n/a	n/a	n/a	Li [162]
	Sichuan (Aba Prefecture)	19	40		Apr–Dec 2008		+	+	+	n/a	n/a	n/a	n/a	Li [163]
	Sichuan (Shiqu County)	3028	3570		Nov 2015–May 2017		n/a	+	+	n/a	n/a	n/a	n/a	Yu [18]
	Sichuan	165			May–Oct 2016		(+)	+	+	n/a	n/a	n/a	n/a	Gao [164]
	TAR (Changdu Prefecture)	4			2001–2005		n/a	+	n/a	+	n/a	n/a	n/a	Feng [34]
	TAR (Dingqing County)	12	5200		2007		(+)	+	+	n/a	n/a	n/a	n/a	Feng [34]
	TAR (Nyingchi City)	5	990		Aug–Oct 2016	Tibetan rural population	+	+	+	n/a	n/a	n/a	n/a	Wang [165]
	Xinjiang	84	4000		1993–2003		+	+	+	+	+	(+)	n/a	Gao [166]
	Xinjiang (Nileke County)	13	6360		2004		+	+	+	n/a	n/a	n/a	n/a	Dingmu [32], Meng [33]
	Xinjiang (Hoboksar Mongol Autonomous County)	2	300		2007		(+)	+	+	n/a	n/a	+	n/a	Wang [167]
	Xinjiang (Hoboksar Mongol Autonomous County)	4	800		Apr–May 2013		n/a	+	+	n/a	n/a	+	n/a	Li 168]
India	Chandigarh^e^	1			n/a	C	+	+	+	n/a	n/a	+	n/a	Nagesh [23]
		1			n/a	C	+	+	+	+	n/a	+	n/a	Shaw [24]
	Maharashtra	1			n/a	C	–	+^f^	–	+^f^	+^f^	+	n/a	Tyagi [25]
	Maharashtra	1			n/a	C	+	+	n/a	+	+	+	n/a	Bhatia [26]
		1			n/a	C	n/a	+	+	+	n/a	+	n/a	Prabhakar [27]
		4 (4)			Mar 2010–May 2016		+	+	+	+	(+)	n/a	n/a	Goja [28]
		3			n/a	C	(+)	+	(+)	(+)	n/a	+	n/a	Bansal [29]
Iran	Razavi Khorasan Province	18 (1)			1997–2012		n/a	+	+	+	+	+	n/a	Maddah [20]
Japan	Hokkaido	373 (14)			1937–1997	R	(+)	(+)	n/a	n/a	n/a	(+)	n/a	Ito [169]
	Hokkaido	424			1937–2003	R	n/a	n/a	n/a	n/a	n/a	n/a	n/a	Oku [170]
		50^b^ (1)			1999–2002	R	(+)	(+)	(+)	(+)	n/a	(+)	n/a	Arai [171], Arai [172]
	Hokkaido	500			1937–2005	R	n/a	n/a	n/a	n/a	n/a	n/a	n/a	Inoue [173]
		109			Apr 1999–2005	R	n/a	n/a	n/a	n/a	n/a	n/a	n/a	Taniguchi [39]
				0.013	2000–2005	R	n/a	n/a	n/a	n/a	n/a	n/a	n/a	Taniguchi [39]
		154 (1)			Apr 1999–Mar 2008	R	n/a	n/a	n/a	n/a	n/a	n/a	n/a	Taniguchi [174]
				0.013	1999–2008	R	n/a	n/a	n/a	n/a	n/a	n/a	n/a	Taniguchi [174]
	Hokkaido	715			1937^a^–2016	R	n/a	n/a	n/a	n/a	n/a	n/a	n/a	Ito [38]
Kazakhstan	Almaty Oblast^c^	46			2006–2014		n/a	+	n/a	n/a	n/a	+	n/a	Abdybekova [82]
	Aqmola and Almaty Oblasts	4			2007–2013	R	n/a	n/a	n/a	n/a	n/a	n/a	n/a	Abdybekova [82]
	Almaty Oblast	6			2012–2015		n/a	+	+	+	+	n/a	n/a	Baimakhanov [175]
Kyrgyzstan	Naryn Oblast (Kochkor District)	92	1970		2000–2007		n/a	n/a	n/a	n/a	n/a	n/a	n/a	Bodoshova [176]
		186			1996–2007	R	n/a	n/a	n/a	n/a	n/a	n/a	n/a	Bodoshova [78]
	Naryn Oblast			7.1	2010–2011	R	n/a	n/a	n/a	n/a	n/a	+	n/a	Usubalieva [16]
	Osh Oblast	122			2000–2013	R	n/a	n/a	n/a	n/a	n/a	+	n/a	Raimkylov [79]
	Osh Oblast	60		6.0	2013		n/a	n/a	n/a	n/a	n/a	+	n/a	
		26			2007	R	n/a	n/a	n/a	n/a	n/a	+	n/a	
		148		2.6	2013		n/a	n/a	n/a	n/a	n/a	+	n/a	
		581			1996–Mar 2016		n/a	+	+	n/a	+	n/a	n/a	Omorov [80]
	Osh Oblast (Alay District)	104^d^	6400^d^		2012		(+)	+	+	n/a	n/a	(+)	(+)	Bebezov [21]
Mongolia		4			2002, 2006, 2007, 2009		(+)	n/a	n/a	n/a	n/a	+	+	Ito [37]
Pakistan	Khyber Pakhtunkhwa	3			2012–2014^a^		n/a	n/a	n/a	n/a	n/a	n/a	+	Ali [30]
South Korea	Gyeongsangnam-do	1			2001	C	+	+	n/a	+	n/a	+	+	Kim [40], Jeong [177]
Taiwan		1 (1)			n/a	C	n/a	+	n/a	+	+	+	n/a	Huang [41]
Tajikistan	Dushanbe^e^	22			2010–2013		n/a	+	+	+	+	n/a	n/a	Ahmedov [83]
Thailand		1 (1)			n/a	C	n/a	+	+	+	n/a	+	n/a	Warnnissorn [42], Limawongpranee [43]
Turkey	Erzurum Province^e^	40			Feb 1987–Dec 2000		(+)	+	+	(+)	(+)	(+)	n/a	Polat [178]
	Southeastern Anatolia	47			1980–2000		(+)	(+)	n/a	n/a	n/a	(+)	n/a	Uzunlar [19]
	Southeastern Anatolia	18		0.49	1980–1990									
	Southeastern Anatolia	29		0.63	1991–2000									
	Southeastern Anatolia	19	0.4		2000									
	Izmir, Afyonkarahisar, Kütahya, Muş, Erzurum Provinces	8			1980–2001		n/a	n/a	n/a	n/a	n/a	+	n/a	Canda [179]
	Diyarbakır Province^e^	47			1980–2002		n/a	n/a	n/a	n/a	n/a	+	n/a	Kılınç [180]
	Erzurum, Ağrı, Kars, Iğdır, Erzincan, Ardahan, Bayburt, Muş Provinces	22			1999–Jul 2004		n/a	n/a	n/a	n/a	n/a	+	n/a	Gündoğdu [181]

*S* serology, *I* diagnostic imaging (includes US, CT, MRI), *US* ultrasonography, *CT* computed tomography, *MRI* magnetic resonance imaging, *HP* histopathology, *DNA* DNA testing/genotyping, *+* positive diagnostic test using the particular method, − negative diagnostic test using the particular method, *n/a* no information on diagnostic investigation using the particular method; *(+)* diagnostic test using the particular method possibly positive in some of the cases, ^a^Additional information obtained by personal communication with the corresponding author of the publication, ^b^case number includes cases allowing a serological diagnosis without additional findings on imaging, ^c^“a number of cases were referred from other regions”, including 8 cases from eastern Kazakhstan, ^d^cumulative prevalence of 4.2% (*n* = 68) [US + , S(+), HP(+), DNA(+)] and 2.2% (n = 36) (US + “and no follow-up”), ^e^location of the institution where all patients have been diagnosed for AE, ^f^imaging of the brain, ^g^total case number (20 cases/221 villagers/1950s–2001s) based on additional questionnaires; C: case report; R: data from an official registry

**Table 2:** Europe

Country	Region	Total case number (*n*)	Prevalence (*n*/10^5^)	Incidence (*n*/10^5^/year)	Period covered by data	Particulars of the population	S	I	US	CT	MRI	HP	DNA	References
Austria		54 (1)			1982–2000	R	(+)	(+)	(+)	(+)	(+)	(+)	n/a	Kern [68]
		65 (2)			1968–2005		+	+	n/a	n/a	n/a	(+)	n/a	Auer [183]
		65			1991–2011		+	+	n/a	n/a	n/a	(+)	(+)	Schneider [44]
		24		0.029	1991–2000		+	+	n/a	n/a	n/a	(+)	(+)	
		28		0.034	2001–2010		+	+	n/a	n/a	n/a	(+)	(+)	
		13		0.158	2011		+	+	n/a	n/a	n/a	(+)	(+)	
	Vorarlberg	22			1991–2011		+	+	n/a	n/a	n/a	(+)	(+)	Schneider [44]
	Vorarlberg	3		0.08	1991–2000		+	+	n/a	n/a	n/a	(+)	(+)	
	Vorarlberg	12		0.32	2001–2010		+	+	n/a	n/a	n/a	(+)	(+)	
	Vorarlberg	7		1.9	2011		+	+	n/a	n/a	n/a	(+)	(+)	
	Tyrol	21			1991–2011		+	+	n/a	n/a	n/a	(+)	(+)	Schneider [44]
	Tyrol	12		0.17	1991–2000		+	+	n/a	n/a	n/a	(+)	(+)	
	Tyrol	5		0.07	2001–2010		+	+	n/a	n/a	n/a	(+)	(+)	
	Tyrol	4		0.56	2011		+	+	n/a	n/a	n/a	(+)	(+)	
Belarus	Gomel Oblast	1			2008		n/a	n/a	n/a	n/a	n/a	+	n/a	Krasavtsev [65]
	Grodno	5			2008–2017		n/a	+	+	(+)	(+)	+	n/a	Prokopchik [64]
Belgium		3			1982–2000	R	(+)	(+)	(+)	(+)	(+)	(+)	n/a	Kern [68]
		13			1999^a^–2003, 2006, 2007, 2010, 2011	R	n/a	n/a	n/a	n/a	n/a	n/a	n/a	Landen [55]
	Liège, Luxembourg, Namur Provinces	22			1999–Feb 2018		+	+	n/a	n/a	n/a	+	(+)	Cambier [184]
Czechia		20 (2)			1998–2014		(+)	+	(+)	(+)	(+)	(+)	(+)	Kolářová [62]
Denmark		1 (1)			n/a		+	+	n/a	+	n/a	n/a	n/a	Laursen [90]
France		260			1982–2000	R	n/a	n/a	n/a	n/a	n/a	n/a	n/a	Bresson-Hadni [185]
		235			1982–2000	R	(+)	(+)	(+)	(+)	(+)	(+)	n/a	Kern [68]
		417		0.026	1982–2009	R	n/a	n/a	n/a	n/a	n/a	n/a	n/a	Grenouillet [46]
		258		0.023	1982–2000	R	(+)	+	(+)	(+)	(+)	(+)	(+)	Piarroux [186]
		66		0.025	Jan 2001–Jun 2005	R	(+)	+	(+)	(+)	(+)	(+)	(+)	Piarroux [186]
		387^b^			1982–2007	R	(+)	(+)	(+)	(+)	(+)	(+)	(+)	Piarroux [187]
		407^b^			1982–2007	R	(+)	(+)	(+)	(+)	(+)	(+)	(+)	Piarroux [188]
	Doubs department			0.7619	1982–2009	R	n/a	n/a	n/a	n/a	n/a	n/a	n/a	Comte [45], Grenouillet [46]
	Haute-Savoie department			0.2329	1982–2009	R	n/a	n/a	n/a	n/a	n/a	n/a	n/a	Comte [45], Grenouillet [46]
		509^b^		0.027	1982–2011	R	(+)	(+)	(+)	(+)	(+)	(+)	(+)	Said-Ali [125]
		575			1982–2013	R	n/a	n/a	n/a	n/a	n/a	n/a	n/a	Charbonnier [52]
		509			Jul 1982–Jun 2012	R	n/a	n/a	n/a	n/a	n/a	n/a	n/a	Chauchet [93]
Germany		132 (6)			1982–2000	R	(+)	(+)	(+)	(+)	(+)	(+)	n/a	Kern [68]
		136 (6)			1994–2004	R	n/a	n/a	n/a	n/a	n/a	n/a	n/a	Kern [189]
		114			2003–2005	R	n/a	(+)	n/a	n/a	n/a	(+)	n/a	Jorgensen [190]
		312			1992–2011		+	+	(+)	(+)	(+)	+	n/a	Grüner [191]
		523^b^	0.64		1992–2016	R	n/a	n/a	n/a	n/a	n/a	n/a	n/a	Schmidberger [51]
	Baden-Württemberg	237^b^	2.18		1992–2016	R	n/a	n/a	n/a	n/a	n/a	n/a	n/a	Schmidberger [51]
	Bavaria	190^b^	1.48		1992–2016	R	n/a	n/a	n/a	n/a	n/a	n/a	n/a	Schmidberger [51]
Greece		1			1982–2000	R	(+)	(+)	(+)	(+)	(+)	(+)	n/a	Kern [68]
Hungary		1			2004	C	+	+	+	+	+	+	n/a	Horváth [192]
		3^b^			2004–2010^a^	R	+	n/a	n/a	n/a	n/a	n/a	n/a	Dezsényi [57]
		1			2012	C	+	+	+	+	+	+	n/a	Dezsényi [57]
Latvia		14			1999–2010	R	(+)	+	+	n/a	n/a	n/a	n/a	Marcinkutė [47]
Lithuania		47			1998–2005	R	(+)	+	(+)	(+)	n/a	n/a	n/a	Marcinkutė [61]
		80			1997–Jul 2006		(+)	(+)	(+)	(+)	n/a	+	n/a	Bružinskaitė [193]
		58			Jun 2003–2007	R	(+)	(+)	(+)	(+)	(+)	(+)	n/a	Strupas [194]
		179			1997–2013	R	n/a	n/a	n/a	n/a	n/a	n/a	n/a	Marcinkutė [47]
				0.54	2013	R	n/a	n/a	n/a	n/a	n/a	n/a	n/a	Marcinkutė [47]
Netherlands		1 (1)			1982–2000	R	(+)	(+)	(+)	(+)	(+)	(+)	n/a	Kern [68]
	Limburg Province	1			2008	C	+^c^	+	n/a	+	+	+	+	van Dommelen [54]
Poland	Warmia-Masuria, Pomerania, Lubusz Provinces	20			Sep 1992–May 2002	R	n/a	(+)	(+)	n/a	n/a	(+)	n/a	Stefaniak [195]
		14			1982–2000	R	(+)	(+)	(+)	(+)	(+)	(+)	n/a	Kern [68]
		6			n/a		(+)	n/a	n/a	n/a	n/a	+	(+)	Myjak [58]
	Warmia-Masuria, Pomerania, Lubusz and Podkarpackie Provinces	45			1992–2006	R	(+)	(+)	(+)	(+)	n/a	(+)	n/a	Stefaniak [196], Stefaniak [197]
		4^b^			2011	R	(+)	(+)	n/a	n/a	n/a	(+)	(+)	Czarkowski [199]
		7^b^			2012	R	(+)	(+)	n/a	n/a	n/a	(+)	(+)	Gołąb [200]
		121^b^		0.014	1990–2011		(+)	(+)	(+)	n/a	n/a	(+)	(+)	Nahorski [49]
	Warmia-Masuria Province	65^b^		0.20	1990–2011		(+)	(+)	(+)	n/a	n/a	(+)	(+)	Nahorski [49]
Romania	Iași, Botoșani, Vaslui Counties	5			Feb 2007–Jun 2007^a^		n/a	n/a	n/a	n/a	n/a	n/a	+	Šnábel [59]
Russia	Kamchatka Krai^d^	9			n/a–2008		n/a	+	+	n/a	n/a	n/a	n/a	Kharchenko [100]
	Sakha Republic^d^	2			2006–2011		n/a	+	+	n/a	+	+	n/a	Sleptsov [99]
		41			2008	R	n/a	n/a	n/a	n/a	n/a	n/a	n/a	Konyaev [98]
	Altai Krai^d^	8			2008	R	n/a	n/a	n/a	n/a	n/a	n/a	n/a	Konyaev [98]
	Tomsk Oblast^d^	42			n/a–2012		n/a	+	+	+	+	n/a	n/a	Kuracheva [66]
		30			2001	R	n/a	n/a	n/a	n/a	n/a	n/a	n/a	Konyaev [13]
	Moscow	1			May 2008–Jun 2014		n/a	+	+	+	n/a	n/a	n/a	Gautier [94]
	Republic of Bashkortostan	1			2014		n/a	n/a	n/a	n/a	n/a	+	n/a	Nartaylakov [96]
	Moscow	5			n/a–2015		n/a	+	n/a	+	+	n/a	n/a	Kotlayrov [95]
	Chelyabinsk Oblast^e^	1			2017		n/a	+	n/a	+	+	n/a	n/a	Zotova [97]
Slovakia	Žilina, Prešov, Košice, Trenčín and Banská Bystrica Regions	10			2000–2007		+	+	+	+	n/a	+	(+)	Kinčeková [201]
	Prešov, Žilina, Košice, Trenčín Regions	16			2000–2010	R	n/a	n/a	n/a	n/a	n/a	n/a	n/a	Miterpáková [60]
	Žilina, Prešov, Košice, Trenčín Regions	26			2000–2013		(+)	(+)	(+)	(+)	(+)	(+)	(+)	Antolová [63]
		37			2000–2014		(+)	(+)	(+)	(+)	(+)	(+)	(+)	Antolová [202]
Slovenia		9^b^	0.45^b^	0.09^b^	2001–2005		+	(+)	(+)	(+)	n/a	n/a	n/a	Logar [50]
Spain	Navarre	1			n/a	C	n/a	+	n/a	+	n/a	+	n/a	Arrechea Irigoyen [67]
Switzerland	Canton of Fribourg	1			1993–1998		+	+	+	+	n/a	n/a	n/a	Gottstein [203]
		118 (6)			1982–2000	R	(+)	(+)	(+)	(+)	(+)	(+)	n/a	Kern [68]
		113			1976–May 2003		n/a	+	n/a	+	n/a	n/a	n/a	Kadry [204]
		60		0.10	1993–2000		+	+	n/a	n/a	n/a	(+)	(+)	Schweiger [48]
		96		0.26	2001–2005		+	+	n/a	n/a	n/a	(+)	(+)	Schweiger [48]
United Kingdom		1 (1)			n/a	C, R^g^	+	+	+	+	+	+	n/a	Graham [22], Kern [68]^g^
		1 (1)			n/a	C	+	+	n/a	+	+^f^	+	n/a	Svrckova [91]

*S* serology, *I* diagnostic imaging (includes US, CT, MRI), *US* ultrasonography, *CT* computed tomography, *MRI* magnetic resonance imaging, *HP* histopathology, *DNA* DNA testing/genotyping, *+* positive diagnostic test using the particular method, − negative diagnostic test using the particular method, *n/a* no information on diagnostic investigation using the particular method; *(+)* diagnostic test using the particular method possibly positive in some of the cases, ^a^Additional information obtained by personal communication with the corresponding author of the publication, ^b^case number includes cases allowing a serological diagnosis without additional findings on imaging, ^c^*Echinococcus* spp. IgG weakly positive; *Echinococcus multilocularis*-specific ELISA negative, ^d^Asian part of Russia, ^e^border region of European and Asian part of Russia, ^f^MRI of the brain; ^g^same case (Petra Kern, personal communication) has also been registered as only case from UK in European Echinococcosis Registry (1982–2000) [70]; C: case report; R: data from an official registry

**Table 3:** Rest of the world

Country	Region	Total case number (*n*)	Prevalence (*n*/10^5^)	Incidence (*n*/10^5^/year)	Period covered by data	Particulars of the population	S	I	US	CT	MRI	HP	DNA	Reference
Canada	Alberta, Ontario, British Columbia, Saskatchewan	12^b^			2001–2014	R	n/a	n/a	n/a	n/a	n/a	n/a	n/a	Massolo [69]
	Alberta, Ontario, British Columbia, Saskatchewan and the Territories^a^	16^b^			2002–2011	R	n/a	n/a	n/a	n/a	n/a	n/a	n/a	Schurer [70]
Morocco		1			n/a	C	+	+	+	+	n/a	+	n/a	Maliki [72]
USA	Minnesota	1			1977	C	+	n/a	n/a	n/a	n/a	+	+	Yamasaki [71]
	City of Chicago	2			2003–2013		(+)	(+)	n/a	(+)	n/a	+	(+)	Taxy [198]

*S* serology, *I* diagnostic imaging (includes US, CT, MRI), *US* ultrasonography, *CT* computed tomography, *MRI* magnetic resonance imaging, *HP* histopathology, *DNA* DNA testing/genotyping, *+* positive diagnostic test using the particular method, *(+)* diagnostic test using the particular method possibly positive in some of the cases, *n/a* no information on diagnostic investigation using the particular method; ^a^Additional information obtained by personal communication with the corresponding author of the publication/Territories (Northwest Territories, Nunavut, Yukon) are coded the same in the registry; ^b^authors assume high probability of non-autochthonous infection; C: case report; R: data from an official registry

**Fig. 4:**
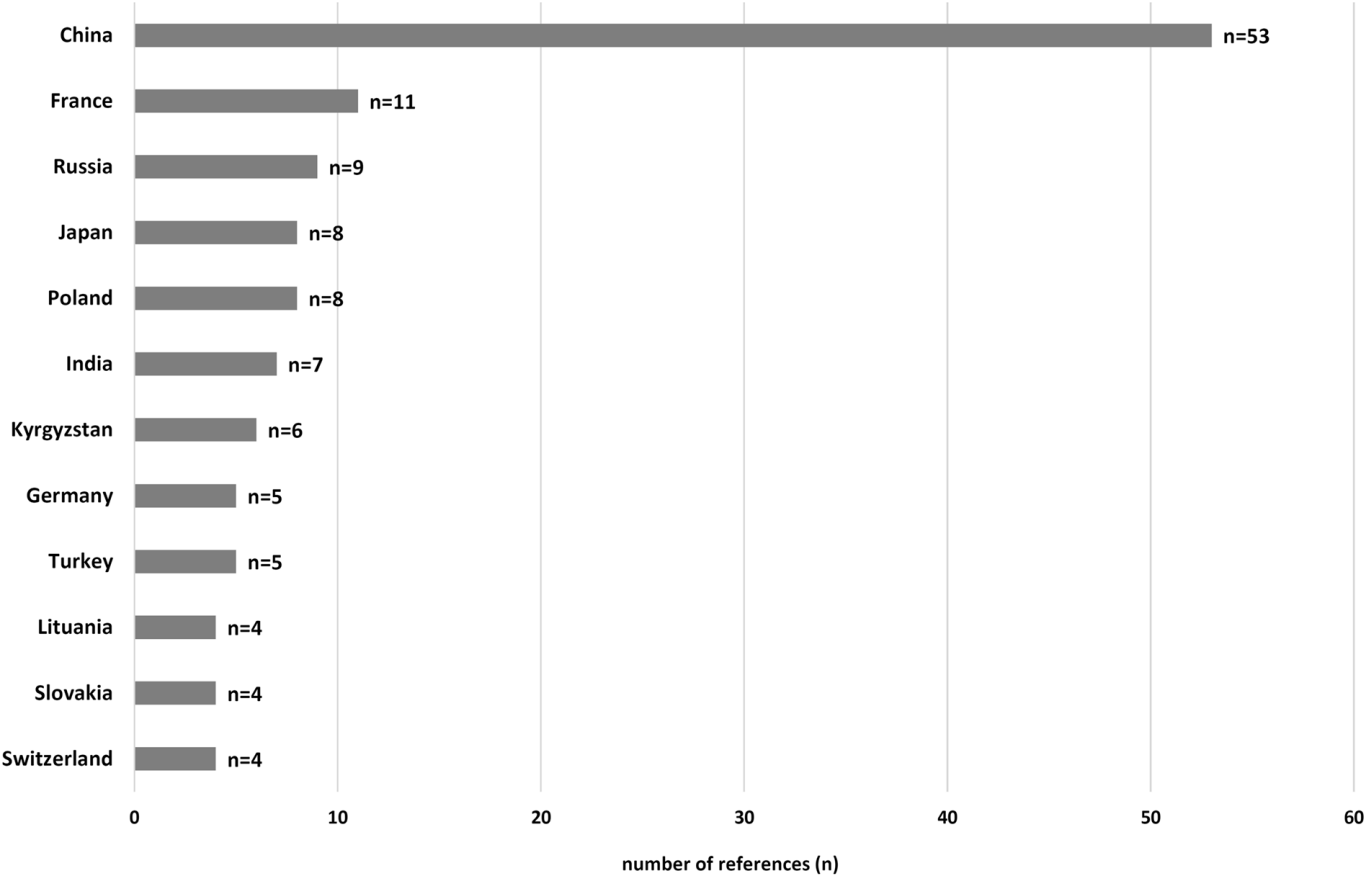
The twelve most frequently listed countries on which epidemiologically relevant papers on AE were published, according to the inclusion and exclusion criteria (data of publication 2001–2018). Redundancy possible

The data obtained from the literature published between 2001 and 2018 showed the presence of human AE in 36 countries within the northern hemisphere. Excluding those countries with a single case report of apparently non-autochthonous origin (United Kingdom, Denmark, Taiwan, and Thailand) leaving a total of 32 countries. In no other country, more epidemiological data were generated than China, with 53 publications, followed by France (*n* = 11), Russia (*n* = 9), Poland (*n* = 8) and Japan (*n* = 8), see also Fig. 4. Apart from two references out of Kyrgyzstan, one from Germany, Slovenia and Turkey, prevalence figures were given only in China (*n* = 47). The incidence was calculated in 14 articles, particularly in France (*n* = 4). Human AE was reported for the first time in this century in Tajikistan, Pakistan, South Korea, Belgium, the Netherlands, Slovakia, Hungary, Lithuania, Latvia, Slovenia, and Morocco, as well as in three countries where the authors considered the cases to be non-autochthonous (Denmark, Taiwan and Thailand).

### Asia

Based on reports from 13 Asiatic countries published since 2001, the epidemiological AE zone stretches across the north of the continent from Turkey to Japan, but with considerable gaps. The highest mean incidence of 7.1/10^5^/year was calculated in Oblast Nary, Kyrgyzstan (2010–2011) [16]. The highest prevalence of 9.43% was found in Banma County in the province of Qinghai, China, in 2014 [17]. The highest absolute number of cases, 3028 patients, came from an US screening in Shiqu County, Sichuan Province (2015–2017) in China [18].

From Turkey, there were five publications with data prior to 2005; in Southeastern Anatolia, the prevalence was calculated to be 0.4/10^5^ in the year 2000 [19]. In central Asia, we found literature from Kazakhstan, Kyrgyzstan and Tajikistan, however no original papers concerning Turkmenistan or Uzbekistan. Reporting the non-autochthonous case from Iran, Maddah and co-authors described the patient as being of Turkmen origin [20]. In the Kyrgyz Alay district, a study has been demonstrated a prevalence of 6.4% in 2012 [21]. From the west of Iran, we found a publication describing 18 cases. There was no literature describing possible Iraqi patients. Noticeably, a paper from the UK showed an Afghan patient who migrated from Pakistan; the authors discuss the infection originating in Afghanistan [22]. There was evidence of isolated cases in India and, as far as we know, the first case was reported in Pakistan [23–30].

Apart from Kyrgyzstan, by far the highest prevalences of human AE were reported in China. Reports have been related exclusively to Western China, namely to the provinces of Qinghai, Gansu, Sichuan and the autonomous regions Xinjiang, Tibet and Ningxia. The highest prevalences were reported from counties of the eastern Tibetan Plateau, ranking up to 9.43% in Banma County (Qinghai, July–August 2014) [17] and 8.5% in Shiqu County (Sichuan, 2001–2002) including a prevalence of 15% in one of the 11 villages being studied [31]. In further administrative divisions of China, the highest prevalences were 6.36% in Xinjiang (2004) [32, 33], 5.2% in Tibet Autonomous Region (TAR) (2007) [34], 3.7% in Ningxia (1985–2001) [35] and 3.39% in Gansu (1996–1997, 2003) [36]. Up north in neighbouring Mongolia, four cases in the west of the country were confirmed by histopathology and molecular genetic testing; the rare Mongolian haplotype was identified in two of these cases [37]. In Japan, it is assumed that nearly all human infections occurred on the northern island of Hokkaido [38]; one paper calculated the mean incidence for the whole of the country to be 0.013/10^5^/year in the period from 2000 to 2005 [39]. The first case from South Korea was reported in a woman who had apparently never left the country [40]. The first AE reports in Taiwan and Thailand were considered to be non-autochthonous by the authors [41–43].

### Europe

In the twenty-first century, AE has been reported in 20 European countries, although the cases in the United Kingdom and Denmark were considered to be non-autochthonous by the authors. A (mean) incidence was calculated in six countries, the highest being in the Austrian Federal State of Vorarlberg in 2011 at 1.9/10^5^/year [44]. The incidence was up to 0.76/10^5^/year in France (in Doubs 1982–2009), up to 0.54/10^5^/year in Lithuania (2013), up to 0.26/10^5^/year in Switzerland (2001–2005), up to 0.20/10^5^/year in Poland (in Warmia-Masuria Province, 1990–2011) and 0.09/10^5^/year in Slovenia (2001–2005) [45–50]. The prevalence was calculated only in a German and a Slovenian study, with the highest German figure of 2.18/10^5^ in the Federal State of Baden-Württemberg (1992–2016) and 0.45/10^5^ for whole Slovenia in the period from 2001 to 2005 [50, 51]. Highest case numbers were registered in the national databases in France (575 from 1982 to 2013) and Germany (523 in 1992–2016) [51, 52]. The first case thought to be autochthonously acquired in the Netherlands was reported in the province of Limburg [53, 54]. The first reported cases from neighbouring Belgium appeared nearly exclusively in Wallonia [55, 56]. Also for the first time in Hungary, a case of AE was thought to be autochthonous in the south-west of the country [57]. Evidence of the disease was found for the first time in Poland and in five patients in north-eastern Romania by molecular genetic testing [58, 59]. To the best of our knowledge, the first reported cases of human AE also originated in Slovakia, Lithuania, Latvia, and Slovenia [47, 50, 60, 61]. Since 2007, 20 cases were registered by the Czech National Reference Laboratory for Tissue Helminthoses [62]. Of the 26 confirmed AE cases in Slovakia between 2000 and 2013, Antolová et al. found that 23 of them occurred in the north-west of the country, in the Žilina and Prešov regions [63]. In Belarus, there were five case reports described in Grodno, a city in the border area to Poland and Lithuania [64], and one post-mortem diagnosis out of Gomel Oblast [65]. From Russia, the highest figure of 42 AE cases was reported in Tomsk Oblast until 2012 [66]. Literature focussing on human AE cases in Ukraine could not be found. In Southern Europe, single cases were documented in the north Spanish province of Navarre and in Greece [67, 68].

### North America

Figures from the Canadian Institute for Health Statistics were published in two articles from Canada, including a total of 12 cases in the southern states of British Columbia, Alberta, Saskatchewan, and Ontario for the period 2001–2014 [69, 70]. In the USA, molecular genetic analysis of the sample from a case reported in Minnesota in 1977 gave a 99.9% agreement in sequence homology with an *E. multilocularis* isolate of a fox in South Dakota and 99.4% agreement with a human sample from Japan [71].

### Africa

In the literature from 2001 to 2018, we found only a single case report of a 54-year-old Moroccan man. This was the first reported case of AE in Morocco [72].

## Discussion

Compared with review articles from around the turn of the millennium [73–77], our work confirms the increasing number of reported cases of human infection in western, northern and eastern Europe, as well as in central Asia. In addition, we found regions in which AE had not been documented before 2001. Even so, there are still fundamental gaps in our knowledge of the distribution of AE.

### Asia

A huge increase in case numbers was seen in central Asia, especially in Kyrgyzstan, the only region in the world where prevalence dimensions were published which otherwise only could be found in China [21, 78]. While only 0–3 cases per annum were recorded in the period 1996–2003, numbers rose continuously from 2004 onwards, reaching 61 cases in 2011 [16]. And more than twice that number of patients was recorded in 2013 (148 cases) [79]. Omorov and Co-Workers collected 1179 AE cases (1996–2015) from nine different Kyrgyz institutions, though diagnostic criteria have been shown for 581 patients [80]. Possible reasons for the upsurge were the improved medical care and diagnostic investigation after the difficult economic period following the dissolution of the Soviet Union and the increasing spread of *E. multilocularis*-infected stray dogs [16, 81]. In Kazakhstan, one hospital in Almaty found that the recorded cases of AE more than doubled from 15 cases in 2004–2011 to 32 cases in 2012–2014 [82]. For the first time, AE cases were published from Tajikistan, where 22 patients were diagnosed from 2010 through 2013 [83]. Only a congress contribution, referring to 83 surgical AE patients missing diagnostic criteria, has been suggested the presence of the disease in Uzbekistan [84].

There is a distinct lack of studies from western Asia, with only estimates of a few AE cases per year existing for Armenia, Georgia and Azerbaijan [5]. Current epidemiological studies out of Turkey are missing. One publication reported 202 AE patients (1980–1998) for the whole country; no diagnostic criteria were given [182]. There are merely sporadic data or none at all from the countries that stretch across central Asia towards China (Iraq, Iran, Afghanistan, Pakistan, and India), so that we have to assume an under-representation [20, 30]. One case report from Iraq suggesting *E. multilocularis* as the causative agent has obviously not been presenting an AE patient [85], as the published CT figure showed the morphologic criteria of a CE lesion (WHO-type 3b) [Tilmann Graeter, personal communication].

By far the highest prevalences, apart from the discussed Kyrgyz numbers, are still to be found in China. Overall, the figures are unchanged and no recent spread, increase, or decrease of case numbers has been described by authors publishing Chinese data. The high prevalence in schoolchildren is particularly remarkable, as paediatric cases of disease are absolute rarities in other parts of the world [86–88].

### Europe

In recent years, there has been both a spread of AE reports across Europe and an increase in case numbers. Until the end of the 1980s, the disease was considered endemic only in the ‘historical’ AE area of western Europe (France, Germany, Switzerland, and Austria) [89], but human infection has already been reported in 20 European countries since 2001. Only the cases in Denmark and the United Kingdom were considered to be non-autochthonous in the respective publications [68, 90, 91]. Figures for all the affected western European countries have shown an increase. In France, according to the FrancEchino Register, the number of new cases per year in the period 2003–2012 (239 cases) almost doubled in comparison with the previous 10 years (122 cases); in particular, there was a significant increase in the incidence of AE in immunosuppressed patients [92, 93]. The mean incidence in Switzerland rose from 0.1/10^5^/year (1993–2000) to 0.26/10^5^/year (2001–2005) [48]. Austria had an average of 2.4 cases in 1991–2000, 2.8 in 2001–2010, and a sudden unexpected rise to 13 cases in 2011 alone [44]. Germany showed a progressive increase in the figures over five-year periods, with 97 cases (2002–2006), 107 cases (2007–2011), and 165 cases (2012–2016) [51]. In addition, AE seems to have spread to previously non-endemic areas such as Belgium and the Netherlands [54–56].

Increasing figures were reported in the literature for all affected eastern European countries. In the five-year periods of the 1990s, the highest number of AE cases detected in Poland was 10, rising to more than 20 cases in 2000–2004, and then over 55 cases in 2005–2009, with a cluster in the north-east of the country bordering Lithuania [49]. Interestingly, five Belarusian case reports were described in a hospital, close to the border area of Poland and Lithuania [64]. In the region of Brest, there are partially unpublished data regarding eight registered AE patients since 1995 (Alla Korzan, personal communication). In Slovakia, there were 11 confirmed cases from 2000 to 2009, but already 15 cases in 2010–2013 [63]. No Czech cases were registered between 1998 and 2006, however 20 cases in the period of 2007–2014 [62]. We can assume the further spread of the disease from the first molecular genetic evidence for the existence of human AE in Romania, as well as the first autochthonous case from south-western Hungary [57, 59]. Despite some high estimated figures of more than 1000 new cases annually [5], there is a distinct lack of data from Russia. Besides numerous single-centre studies with preselected study groups undergoing surgery, epidemiological data could only be found from Moscow [94, 95], the south of the Volga and Ural Federal Districts (neighbouring Republic of Bashkortostan and Chelyabinsk Oblast) [96, 97], the southwest of Siberia Federal District (Tomsk Oblast, Altai Krai) [66, 98] and the Far East Sakha Republic and the peninsula of Kamchatka [99, 100]. Nevertheless, the official registry of the Russian federal agency Rospotrebnadzor reported higher figures in 2008 (41 cases) than in 2001 (30 cases) [13, 98].

Knapp and co-workers studied the genetics of the observed spread to eastern Europe. They used the EmsB microsatellite marker to analyse the genetic diversity of *E. multilocularis* in various European endemic areas. They found the lowest diversity in Slovakia and Poland and the highest in Switzerland and the Swabian Jura, arguing for the two latter regions being the oldest endemic areas in Europe in evolutionary terms and for a ‘mainland-island’ system governing pathogen transmission [101]. In northern Europe, the number of cases in Lithuania rose: the incidence increased from 0.03/10^5^/year in 2004 to 0.57/10^5^/year in 2009 and 0.74/10^5^/year in 2012, exceeding all the overall national incidences in Europe published since 2001 [47]. Six Swedish AE patients with assumed infection abroad were officially reported in the Public Health Agency in 2014 and 2017, with the first two diagnoses in 2012 [102, 103]. The European Food Safety Authority registered three cases in Estonia in 2013 (no information about site of infection or diagnostic criteria) [104]. In Finland, there are unpublished data about a native patient with PCR-confirmed AE and a travel history to endemic destinations in Europe (Antti Lavikainen, personal communication) [105].

In Southern Europe, Slovenian AE cases have been published for the first time. Although there have already been cases of the disease reported in Spain, *E. multilocularis* has never been demonstrated in the wildlife to date, so there is not sufficient evidence for the endemicity of the parasite [67, 106]. In Greece, a patient of Macedonian origin living in Thessaloniki was registered via the European Echinococcosis Registry (Petra Kern, personal communication) [68]. Since its reporting in the late 1990s, no new cases were documented out of this area. In summary, we can say that without exception, we found an increase in reported case numbers in all significant endemic European countries (i.e. countries from which there are at least 20 reported cases of AE). There are three relevant hypotheses to explain this rise in Europe.

First of all, there is an increase in the red fox population in Europe, which is also related to the elimination of rabies, together with higher infection rates with *E. multilocularis* [63, 106, 107]. One Swiss study showed a direct correlation between the growing fox population and the increase in human echinococcosis [43]. The observed increasing urbanisation of the fox habitat is also noteworthy [108–110]. Secondly, improved awareness of AE in the general population and healthcare workers may also have contributed to more cases being diagnosed or fewer incorrect diagnoses being made [111]. Thirdly, the possibilities for diagnostic investigation have improved considerably in the last 20 years, particularly with respect to imaging and molecular genetics [8, 63].

If we compare our findings with the current literature on the distribution of *E. multilocularis* in European red foxes, there are several countries in which the parasite has been detected in red foxes in this century but not in humans (Serbia, Croatia, Italy, Luxembourg, and Ukraine) [106, 112]. Human cases in these countries probably have to be reckoned with in the future.

### North America

AE is extremely rare in North America, even though the infection rates recorded in animals are relatively high, e.g. 44.6% of foxes in northeast Nebraska and 35.3% of coyotes in Illinois [113, 114]. Despite these high figures, only two new cases have reliably been diagnosed in humans in the USA since more than four decades [198]. However, the literature was supplemented by two native individuals from the states of Alaska and Washington who were registered as deaths to AE on a death certificate in the National Center for Health Statistics (NCHS) and, therefore, convincing evidence is still lacking [115, Ben Bristow, personal communication]. From Alaska, where 54 human infections have been reported from 1947 to 1986 [reviewed by 116], no confirmed subsequent cases were found. In Canada, AE cases are thought to be (predominantly) non-autochthonous [69, 70]. Interestingly, a public health report of the government of the province of Alberta suggests some diagnosed autochthonous cases since 2013 [117]. Furthermore, the Canadian Institute for Health Information has documented at least three human infections in Ontario between 2014 and May 2018 [118] and a recent review of Wen and co-workers has mentioned unpublished case reports in Quebec and Manitoba [119]. Besides the possibility of misdiagnosis, one hypothesis to explain the discrepancy between the high infection rates in the wildlife and the extreme rarity of autochthonous cases in North America is the genetic expression of *E. multilocularis* in these areas combined with a human population of correspondingly low susceptibility. To the best of our knowledge, in fact, there has been only one proven case of human infection with the North American haplotype so far, detected in a DNA-based analysis of a sample of a patient diagnosed in Minnesota in 1977 [12, 120]. Remarkably, since 2009, multiple cases of animals infected with the European-type strain have been documented in Canada, including dogs infected as intermediate hosts. Unlike the North American strain, this strain is typically associated with human disease. We can, therefore, speculate that the European strain could have become established in the region and any human cases in Canada in the future may indeed be autochthonous in nature [121, 122, Janna Schurer, personal communication].

### Africa

As with the case in Spain, despite the transparency of the diagnostic investigations in the case report from Morocco, *E. multilocularis* has not been confirmed in North African wildlife and there is not yet any concrete evidence of its presence [5, 8].

### Limitations

On the basis of the available literature, it is currently not possible to obtain a valid list of the worldwide prevalence of human AE without many gaps, as both the quantity and quality of the published data are insufficient for the purpose. The reasons for this are manifold. AE is a notifiable disease only in some places, e.g. in most European countries but, on the North American continent, only in the Northwest Territories and Ontario in Canada [105, 111, 118]. AE is frequently not distinguished from CE, even though the latter is a distinct disease entity with different transmission profiles, risk factors, and clinical manifestations, requiring quite different control and surveillance measures and treatment [1, 123]. Out of the echinococcosis cases officially notified to the European Union in 2013, 31.7% (253 cases) did not differentiate between AE and CE [124]. Furthermore, due to the initial asymptomatic period of 5–15 years, it is usually not possible to pinpoint the precise location where the parasite was ingested [75]. In addition, most entries in the registries do not have a case definition, i.e. are not based on firm diagnostic criteria, which makes it more difficult to compare the recorded data. If there was a case definition given by the authors, we could not verify its quality by reviewing the defining imaging data or other diagnostic criteria.

Because of the long incubation period, we have to suspect the existence of a large proportion of asymptomatic individuals, who have also not been recorded. There are probably also considerable numbers of symptomatic patients living in poor economic and/or remote areas who remain undiagnosed and are therefore not included in epidemiological registries or corresponding studies [8, 75].

One limitation of this article is the lack of comparability of the units in the data. The prevalence or incidence is only rarely calculated in the scientific papers, which makes the interpretation of case numbers more challenging.

Another limitation can be found in the fact that, although the literature was published in 2001–2018, the time frame of the diagnosed cases ranged from 1937 up to 2017, giving the data a temporal inhomogeneity. Even though an increase of reported cases as well as a spread in several mentioned areas has been confirmed by this work, a significant bias cannot be excluded; a statistical analysis did not seem reasonable due to the inhomogeneity of the collected data. Due to recent improvement of diagnostics, as discussed above, an increase in data quantity can be assumed [8, 63].

Following our inclusion and exclusion criteria, it can be supposed that grey literature of interest is missing in our synopsis. We discussed some of these sources above; adding those cases from Uzbekistan, Sweden, Finland and Estonia, AE could be assumed to have been reported in overall 40 countries in the twenty-first century. However, only in 36 of those nations, publications which meet some basic quality criteria were existent.

With respect to the mapping, the main limitation is the worldwide lack of valid data, insufficient even for topographical interpolation estimating the borders of endemic disease areas and the corresponding prevalences.

From the epidemiological point of view, a national obligation to report AE as a notifiable disease, including its differentiation from CE, would be desirable in endemic countries. The data should be entered into national AE registries, which should be standardised and coordinated on an international level to generate comparable datasets and ultimately ensure high validity [125]. In addition, every effort should be made to achieve a precisely defined uniform terminology relating to echinococcosis and its pathogenic agents.

## Conclusions

This systematic review provides an overview of the epidemiologically relevant literature on AE in the twenty-first century and underlines trends in the distribution of human AE. Our paper demonstrates an increasing number of reported cases in western, northern and eastern Europe, as well as in central Asia. In addition, we have established areas in which AE was not reported prior to 2001. The study shows that there are still fundamental gaps in our knowledge on the endemicity of the disease, as well as its prevalence and incidence. Original studies on the prevalence and incidence of AE are lacking from many parts of the world and further research on the subject is required.

## Abbreviation

SB: Sven Baumann
RS: Rong Shi
WL: Wenya Liu
HB: Haihua Bao
JS: Julian Schmidberger
WK: Wolfgang Kratzer
WXL: Weixia Li
