# Correction to: Worldwide literature on epidemiology of human alveolar echinococcosis: a systematic review of research published in the twenty-first century

**DOI:** 10.1007/s15010-021-01695-6

**Published:** 2021-10-11

**Authors:** Sven Baumann, Rong Shi, Wenya Liu, Haihua Bao, Julian Schmidberger, Wolfgang Kratzer, Weixia Li, the interdisciplinary Echinococcosis Working Group Ulm, Thomas F. E. Barth, Sven Baumann, Johannes Bloehdorn, Iris Fischer, Tilmann Graeter, Natalja Graf, Beate Gruener, Doris Henne-Bruns, Andreas Hillenbrand, Tanja Kaltenbach, Peter Kern, Petra Kern, Katharina Klein, Wolfgang Kratzer, Niloofar Ehteshami, Patrycja Schlingeloff, Julian Schmidberger, Rong Shi, Yael Staehelin, Frauke Theis, Daniil Verbitskiy, Ghaith Zarour

**Affiliations:** 1 Department of Internal Medicine I, Ulm University Hospital, Albert-Einstein-Allee 23, 89081 Ulm, Germany; 2 Department of Diagnostic and Interventional Radiology, Ulm University Hospital, Albert-Einstein-Allee 23, 89081 Ulm, Germany; 3 Xinjiang Medical University, First Affiliated Hospital, WHO Collaborating Centre on Prevention and Care Management of Echinococcosis, Urumqi, 830000 Xinjiang Uyghur Autonomous Region People’s Republic of China; 4 Qinghai University Affiliated Hospital, Qinghai University, Xining, 810001 Qinghai People’s Republic of China

## Correction to: Infection (2019) 47:703–727 10.1007/s15010-019-01325-2

**Acknowledgements** Members of the Echinococcosis Working Group Ulm: Thomas FE Barth, Sven Baumann, Johannes Bloehdorn, Iris Fischer, Tilmann Graeter, Natalja Graf, Beate Gruener, Doris Henne-Bruns, Andreas Hillenbrand, Tanja Kaltenbach, Peter Kern, Petra Kern, Katharina Klein, Wolfgang Kratzer, Niloofar Ehteshami, Patrycja Schlingelof, Julian Schmidberger, Rong Shi, Yael Staehelin, Frauke Theis, Daniil Verbitskiy, Ghaith Zarour.

The original version of this article unfortunately contained a mistake. Figure 3 in the original version of this article has been replaced. The corrected Fig. 3 is given below.

**Fig. 3:**
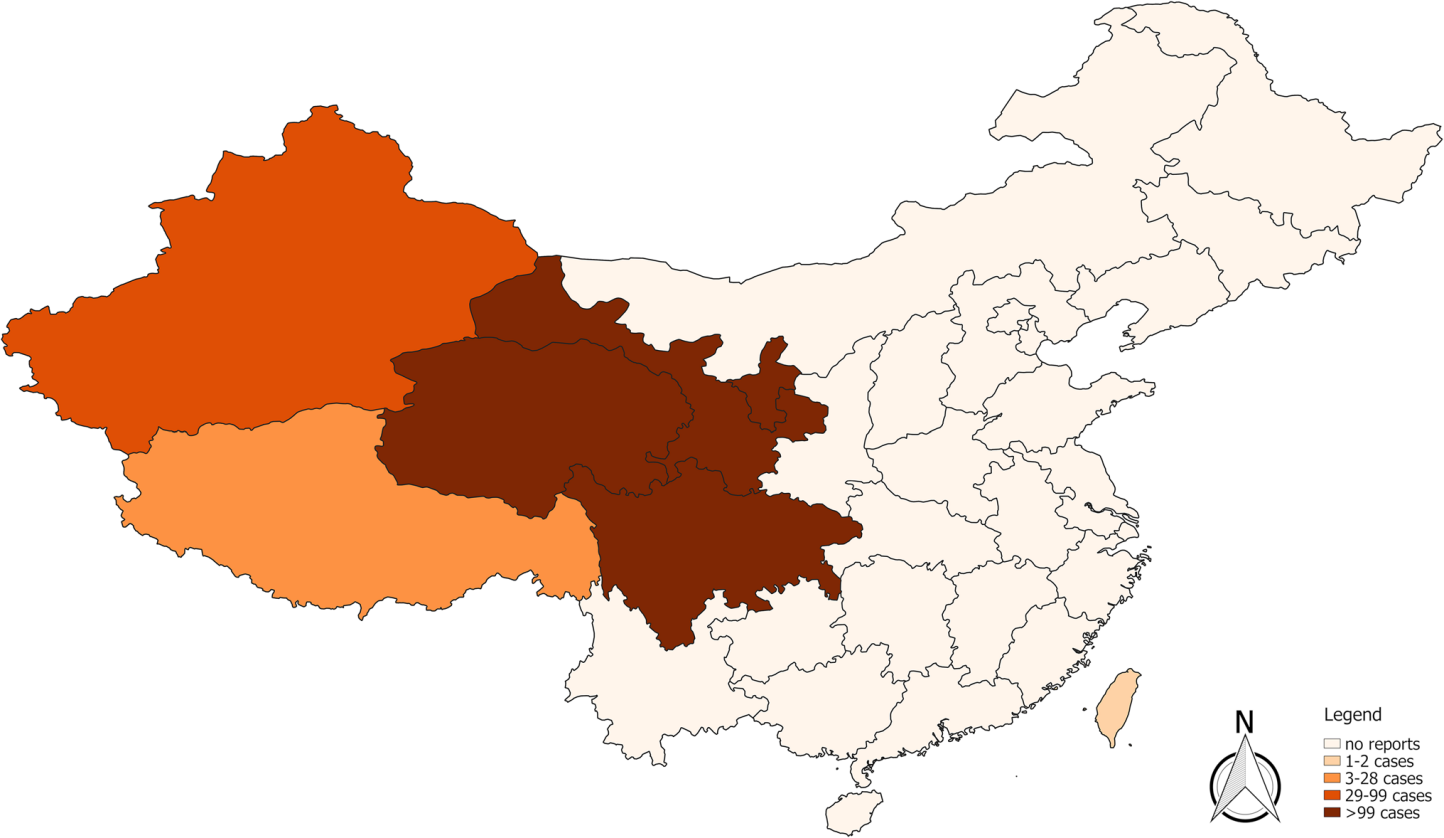
Current distribution of alveolar echinococcosis in humans according to the published literature 2001–2018 in China. Each province in which cases of AE had been reported in the literature between 2001 and 2018 was mapped. For the topographical colour shading of a province, the highest total number of cases in one reference within this period was the deciding factor

The original article has been corrected.

